# Leaf pigmentation, its profiles and radical scavenging activity in selected *Amaranthus tricolor* leafy vegetables

**DOI:** 10.1038/s41598-020-66376-0

**Published:** 2020-10-29

**Authors:** Umakanta Sarker, Shinya Oba

**Affiliations:** 1 Department of Genetics and Plant Breeding, Faculty of Agriculture, Bangabandhu Sheikh Mujibur Rahman Agricultural University, Gazipur, 1706 Bangladesh; 2 Laboratory of Field Science, Faculty of Applied Biological Sciences, Gifu University, Yanagido 1-1, Gifu, Japan

**Keywords:** Natural variation in plants, Plant breeding

## Abstract

The selected *A. tricolor* accessions contained abundant color attributes, betacyanin, carotenoids, betalains, betaxanthins, and antioxidants potentiality that varied in terms of genotypes. For the first time, we identified 4 betacyanins, and 5 carotenoid compounds in *A. tricolor* genotypes. The genotype VA14 and VA16 had abundant color attributes, betacyanin such as amaranthine, iso-amaranthine, betanin, iso-betanin, and antioxidants potentiality. These two genotypes having an excellent source of color attributes, betacyanins, betalains, betaxanthins, and antioxidants potentiality could be used as potent antioxidant varieties. The genotype VA11 and VA16 had abundant carotenoid components, such as zeaxanthin, lutein, violaxanthin, neoxanthin, total xanthophylls, and beta-carotene. The genotype VA11 and VA16 had abundant carotenoid components that could be used as carotenoid enrich varieties. It revealed from the correlation study that pigment profiles of *A. tricolor* genotypes exhibited high quenching capacity of radicals. These accessions have high antioxidant potentials and great opportunity to make drinks, preservatives, and colorant of food products to feed the community deficient in antioxidants. The identified components of betacyanins and carotenoids in *A. tricolor* require comprehensive pharmacological study. The baseline data on color attributes, betacyanins profile, carotenoids profile, betaxanthins, betalains and antioxidant potentiality obtained in the present study could contribute to pharmacologists for evaluating these components scientifically in *A. tricolor*.

## Introduction

Edible stems and leaves of *Amaranthus* are an inexpensive and abundant source of digestive fiber, protein containing methionine and lysine, vitamin C, carotenoids, minerals^1–6^. It has also abundant antioxidant pigments, such as betacyanin, anthocyanin, betaxanthin, betalain, carotenoids, and chlorophylls^7–10^; bioactive phytochemicals including ascorbic acid, flavonoids, and phenolic acids^11–14^. These bioactive components of natural origin have a remarkable contribution to the industry of food as these quench reactive oxygen species (ROS) and cure many diseases including cardiovascular diseases, arthritis, cancer, cataracts, retinopathy, atherosclerosis, emphysema, and neurodegenerative diseases^15–18^.

The acceptability of foods mostly depends on color, flavor, and taste. For these reasons, recently coloring products of foods become very popular due to meet up the general interest of the people around the globe. These products meet up the general interest of consumers in the safety, nutritional and aesthetic aspects of foods. It meet up the requirement for betacyanin, anthocyanin, carotenoids, and chlorophylls. *Amaranthus* leafy vegetable is a sole source of betacyanins including amaranthine, iso-amaranthine, betanin, iso-betanin that has important scavenging activity of free radicals^19^. Betacyanin could be used as a food colorant in low-acid foods. It has higher pH stability than anthocyanins^20^. Amaranthine, a major pigment of betacyanin in amaranth had very strong quenching capacity of radicals. It is an alternate source of betanins (red beets) for colorant of foods and potential antioxidants^19^. It is widely adapted to abiotic stresses including drought^21–24^ and salinity^25–27^. Red color amaranth has more pigments, such as betacyanin and carotenoids than green color amaranth. Amaranth leaves inhibited the proliferation of colon (Caco-2) cancer cell lines, breast (MCF-7) cancer cell lines, liver (HepG2) and exhibited anticancer potential^28^.

In many developing countries including Bangladesh and India, deficiency of vitamin A and macular degeneration associated with aging are serious threats among adults and children. In developing countries, age-related macular degeneration is increasing at an alarming rate. Consumption of inadequate macular pigments and pro-vitamin A in regular diet is the main cause of deficiency of vitamin A and macular degeneration associated with aging. So, regular consumption of vitamin A enriches vegetables may be the solution of deficiency of vitamin A and macular degeneration associated with aging. *A. tricolor* is the inexpensive natural source of pro-vitamin A due to high content of beta-carotene and xanthophylls profile including lutein, violaxanthin, neoxanthin, and zeaxanthin. The carotenoids profile of crops varies markedly in different climatic conditions. Carotenoids profile of the same species may vary due to the difference in eco-geographical regions. Recently, we have been exploring the possibility of pigments and phenolic profiles of *A. tricolor* including antioxidant potentials as it has abundant natural betacyanins and carotenoids profile containing macular pigments along with phenolic profiles, antioxidant constituents, and antioxidant activities of interest in the industry of foods^17,29^. In our earlier studies, we selected a few high yields and antioxidant potential *A. tricolor* genotypes from 43 germplasms. It is the first attempt to study the color attributes, betacyanin, carotenoids, and phenolic profiles, antioxidant constituents, and antioxidant potentials in selected *A. tricolor* elaborately through high performance liquid chromatograph. Therefore, we ultimately evaluate the possibility of selection of appropriate genotypes for extracting colorful juice as drinks, preservatives, and colorant of food products containing abundant betacyanin, carotenoids, and phenolic profiles, antioxidant constituents, and antioxidant potentials.

## Results and discussion

### Color attributes

**Figure 1:**
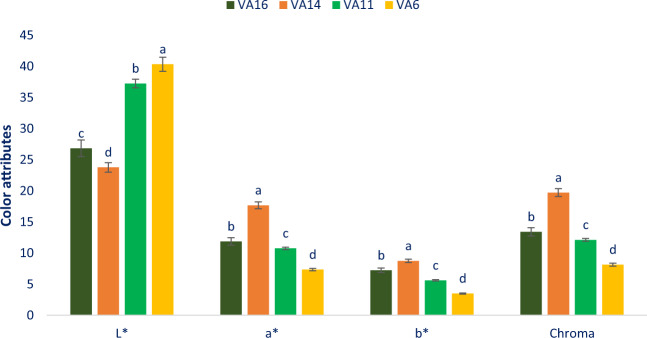
Color attributes in four selected *A. tricolor* leafy vegetables, L*, Lightness; a*, Redness/greenness; b*, Yellowness/blueness, different letters in the bar are differed significantly by Duncan Multiple Range Test ((P < 0.01), (n = 3).

Figure 1 shows the leaf color attributes of four selected *A. tricolor*. The pronounced variability was noted with regard to b*, lightness (L*), a*, and chroma of the four accessions studied. The range of chroma, a*, b*, and lightness (L*) were 8.15 to 19.73, 7.35 to 17.68, 3.51 to 8.75, and 23.78.34 to 40.35, respectively. The maximum values of lightness was noticed in VA6 (40.35), while the minimum values of lightness was reported in VA14 (23.78) followed by VA16 (26.84). Similarly, the maximum a* (17.68), chroma (19.73), and b* (8.75) value were noted in VA14 followed by VA16, while the lowest a* (7.35), b* (3.51), and chroma (8.15) were found in VA6. We found corroborative findings with the findings of Colonna *et al*.^30^ and Sarker and Oba^31^. Color attributes are essential parameters that have contributed significantly to the choice of consumers, their acceptability and preferability. These are main attributes for testing the potentiality of antioxidants in vegetables^30^. Deep red accessions VA14 and VA16 had high values of yellowness and redness representing the occurrence of plentiful pigments including betaxanthin, anthocyanins, betacyanin, and betalain. Conversely, the accession VA6 exerted low values of yellowness and redness representing the occurrence of low pigments including betaxanthin, anthocyanins, betacyanin, and betalain. The four bright red color accessions have ample betacyanin pigments showing better stability at pH 5–7 and lower temperatures (<14 °C)^32^. These accessions have great opportunity to make drinks, preservatives and colorant of food products.

### Betacyanin components

**Figure 2:**
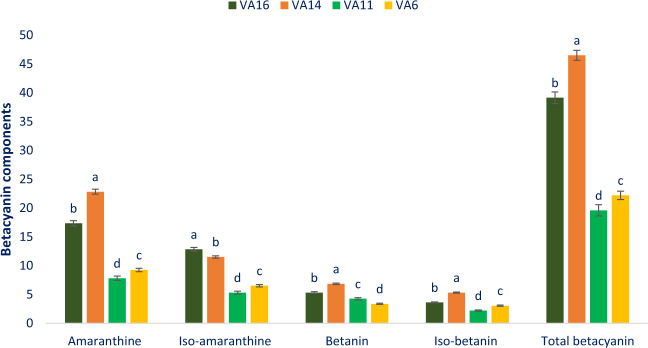
Betacyanin profiles in four selected *A. tricolor* leafy vegetables, Amaranthine (mg 100 g^−1^), Iso-amaranthine (mg 100 g^−1^), Betanin (mg 100 g^−1^), Iso-betanin (mg 100 g^−1^), Total betacyanin (mg 100 g^−1^), different letters in the bar are differed significantly by Duncan Multiple Range Test ((P < 0.01), (n = 3).

The betacyanin is responsible for the red-violet and maroon color in the appearance of *A. tricolor* genotypes. Table 1 shows main fragment ions (MS^2^), molecular ion, retention time, λmax, and detected betacyanin components. The liquid chromatograph separated values of betacyanin components from four genotypes (VA16, VA14, VA11, and VA6) were compared with standard betanin (Betanin from red beet were used as standards) using the respective peaks of the compounds. Figure 2 represents the individual betacyanin components and their relative proportions in the extracts. The HPLC determined betacyanin exhibited 2 principal peaks (peak1 and peak2) at 538 nm (Table 1). Peak1 and peak2 were eluted before the peaks of betanin and iso-betanin standards (peak3 and peak4), representing a greater intensity of glycosylation^33^. The betacyanin components amaranthine and iso-amaranthine were abundant pigments in *Amaranthus*^34^. Cai *et al*.^35^ also reported that amaranthine as well as iso-amaranthine (C15 epimer of amaranthine) were widely distributed and available (91.5%) in forty accessions of thirty seven species of eight genera of *Amaranthus*. Betacyanin components amaranthine and betanin have betanidin (unit of aglycone). Betanin has glucoside in lieu of glucuronosylglucoside of amaranthine. Both peak3 and peak4 represented the molecule at *m/z* 551 [M + H]^+^, matching the mass of glucoside of betanidin, and their aglycone ion (main fragment ion) were at *m/z* 389 [M-glucose + H]^+^. In *A. tricolor*, both peak1 and peak2 represented the molecule at *m/z* 727 [M + H]^+^, corresponding to glucuronosylglucoside of betanidin. Both peak1 and peak2 represented the major fragment ions at *m/z* 551 [M-glucuronic acid + H]^+^, and with the same aglycone ion at *m/z* 389 [M-glucuronosylglucose + H]^+^ (Table 1). Based on the UV–Vis and MS data presented in Table 1 and those obtained by others, peak1 and peak2 were tentatively identified as amaranthine and iso-amaranthine (C15 epimer), respectively^32,33^.

**Table 1:** Retention time (Rt), wavelengths of maximum absorption in the visible region (λ_max_), mass spectral data and tentative identification of betacyanin components in four selected *A. tricolor* leafy vegetables.

Peak no	Rt (min)	λ_max_ (nm)	Molecular ion [M - H]^-^ (m/z)	MS^2^ (m/z)	Identity of tentative betacyanin component
1	1.45	538*	727	551, 389	Amaranthine (betanidin 5-*O*-β-glucuronosylglucoside)
2	1.75	538*	727	551, 389	Iso-amaranthine (isobetanidin 5-*O*-β-glucuronosylglucoside)
3	2.15	538*	551	389	Betanin (betanidin 5-*O*-β-glucoside)
4	2.46	538*	551	389	Iso-betanin (isobetanidin 5-*O*-β-glucoside)

^*^ Stintzing *et al*.^33^.

Considering betacyanin components, amaranthine was the most abundant betacyanin followed by iso-amaranthine in *A. tricolor* genotypes (Fig. 2). Our obtained amaranthine, iso-amaranthine, betanin and iso-betanin content in *A. tricolor* genotype VA14 and VA16 were greater than the results of Stinzing *et al*.^33^ in *A. spinosus* stem. Amaranthine, iso-amaranthine, betanin, iso-betanin, and total betacyanin ranged from 7.82 to 22.84, 5.32 to 12.83, 3.37 to 6.84, 2.21 to 5.32, and 19.60 to 46.52 mg 100 g^−1^, respectively (Fig. 2). The highest amaranthine (22.84 mg 100 g^−1^), betanin (6.84 mg 100 g^−1^), iso-betanin (5.32 mg 100 g^−1^) and total betacyanin (46.52 mg 100 g^−1^) were obtained from the genotype VA14 followed by the genotype VA16. Cai *et al*.^19^ also reported that amaranthine was the major compounds in betacyanin components in the plants of Amaranthaceae which corroborated with the present findings. Whereas, the genotype VA16 showed the highest iso-amaranthine (12.83 mg 100 g^−1^) followed by VA14. In contrast, the genotype VA11 showed the lowest amaranthine (7.82 mg 100 g^−1^), iso-amaranthine (5.32 mg 100 g^−1^), iso-betanin (2.21 mg 100 g^−1^) and total betacyanin (19.60 mg 100 g^−1^) and the genotype VA6 exhibited the lowest betanin (3.37 mg 100 g^−1^). Within leafy vegetables, amaranth is a unique and excellent source of betacyanin components including amaranthine, iso-amaranthine, betanin, iso-betanin. These betacyanin components have high scavenging activity of free radicals and act as an excellent antioxidants^19^. Betacyanin components could be used as a food colorant in low-acid foods as these components have higher pH stability compared to anthocyanins^20^. Amaranthine, a major pigment of betacyanin components in amaranth had very strong quenching capacity of radicals and also act as an excellent antioxidants. It is an alternate source of betanins (red beets) for colorant of foods and potential antioxidants^19^. Hence, the genotypes VA14 and VA16 containing high betacyanin profiles including amaranthine, iso-amaranthine, betanin, iso-betanin could be used for extracting colorful juice as drinks, preservatives, and colorant of food products.

### Carotenoid profiles

**Figure 3:**
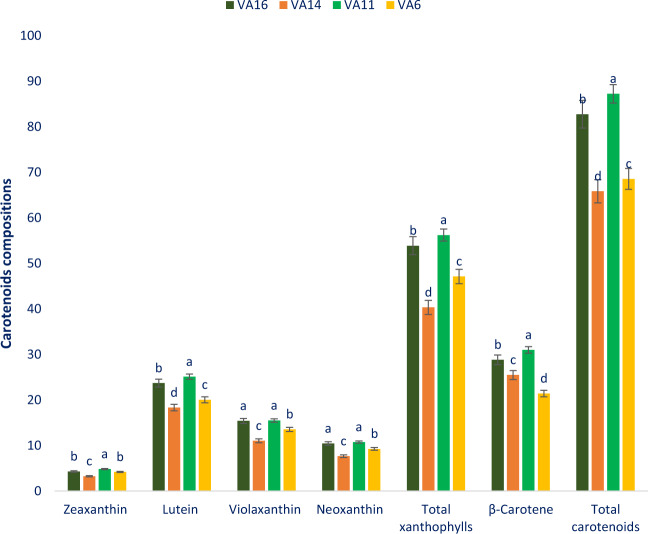
Carotenoid profiles (mg 100 g^−1^ FW) in four selected *A. tricolor* leafy vegetables, different letters in the bar are differed significantly by Duncan Multiple Range Test ((P < 0.01), (n = 3).

Table 2 represents the data on the molecular ion, main fragment ions in MS^2^, λmax, retention time, and identified carotenoid compounds. The LC separated carotenoid values from four genotypes (VA16, VA14, VA11, and VA6) were compared with standard carotenoid compounds masses through the corresponding peaks of the compounds. A total of five carotenoid compounds were identified in *A. tricolor*. Among them, four compounds were xanthophylls (Lutein, violaxanthin, neoxanthin, and zeaxanthin) and one compound was pro-vitamin A (beta-carotene). Figure 3 represents the identified carotenoid profiles along with total xanthophylls and total carotenoids of four selected *A. tricolor* leaves, respectively.

**Table 2:** Retention time, wavelengths of maximum absorption in the visible region (λ_max_), mass spectral data and tentative identification of carotenoid profiles in four selected *A. tricolor* leafy vegetables.

Peak no	Retention time (min)	λ_max_ (nm)	Molecular ion [M - H]^-^ (m/z)	MS^2^ (m/z)	Identity of tentative carotenoids
1	2.4	450	438.48	438.47	Neoxanthin
2	2.6	450	446.38	446.39	Violaxanthin
3	3.8	450	445.19	445.16	Lutein
4	4.2	450	452.59	452.56	Zeaxanthin
6	20.2	450	449.47	449.48	Beta-carotene

Within xanthophylls, lutein was identified as the most abundant carotenoids followed by violaxanthin and neoxanthin while the content of zeaxanthin was very low in *A. tricolor* genotypes (Fig. 3). Except for violaxanthin, we obtained much greater lutein, neoxanthin, zeaxanthin, total xanthophylls, beta-carotene, and total carotenoid contents in *A. tricolor* genotype compared to the contents of *A. gangeticus* genotype of Raju *et al*.^36^. There were no prominent variations in zeaxanthin, lutein, violaxanthin, neoxanthin, total xanthophylls, beta-carotene, and total carotenoids content of the studied genotypes, albeit those showed the significant differences among studied genotypes. Zeaxanthin, lutein, violaxanthin, neoxanthin, total xanthophylls, beta-carotene, and total carotenoids ranged from 3.28 to 4.84, 18.34 to 25.12, 11.05 to 15.51, 7.67 to 10.77, 40.34 to 56.24, 21.43 to 31.01, and 65.85 to 87.25 mg 100 g^−1^, respectively (Fig. 3). The highest zeaxanthin (4.84 mg 100 g^−1^), lutein (18.34 mg 100 g^−1^), violaxanthin (15.51 mg 100 g^−1^), neoxanthin (10.77 mg 100 g^−1^), total xanthophylls (56.24 mg 100 g^−1^), and total carotenoids (87.25 mg 100 g^−1^) were obtained from the genotype VA11 followed by the genotype VA16. In contrast, the genotype VA14 showed the lowest zeaxanthin (3.28 mg 100 g^−1^), lutein (18.34 mg 100 g^−1^), violaxanthin (11.05 mg 100 g^−1^), neoxanthin (7.67 mg 100 g^−1^), total xanthophylls (40.34 mg 100 g^−1^), and total carotenoids (65.85 mg 100 g^−1^). The genotype VA11 exhibited the highest beta-carotene (31.01 mg 100 g^−1^), followed by the genotype VA16. In contrast, the genotype VA6 showed the lowest beta-carotene (21.43 mg 100 g^−1^) (Fig. 3).

**Figure 4:**
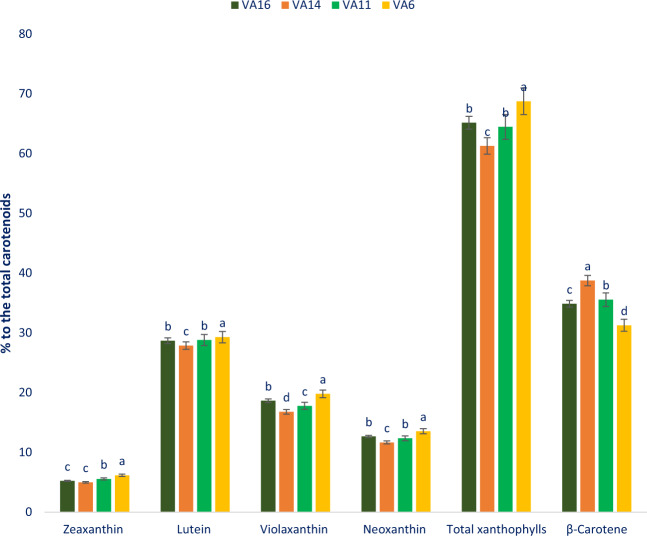
Percentage of zeaxanthin, lutein, violaxanthin, neoxanthin, total xanthophyll and beta-carotene to the total carotenoids in four selected *A. tricolor* leafy vegetables, different letters in the bar are differed significantly by Duncan Multiple Range Test ((P < 0.01), (n = 3).

Percentage of zeaxanthin, lutein, violaxanthin, neoxanthin, total xanthophylls, beta-carotene to total carotenoids ranged from 4.98 to 6.15, 27.85 to 29.27, 16.78 to 19. 79, 11.65 to 13.53, 61.26 to 68.75, and 31.25 to 38.74 mg 100 g^−1^ FW, respectively (Fig. 4). The genotype VA6 demonstrated the highest percentage of zeaxanthin, lutein, violaxanthin, neoxanthin, total xanthophylls to total carotenoids followed by VA16 and VA11 albeit this genotype exhibited the lowest beta-carotene. In contrast, VA14 showed the lowest percentage of zeaxanthin, lutein, violaxanthin, neoxanthin, total xanthophylls albeit this genotype exhibited the highest beta-carotene. It indicated from the results that the percentage of total xanthophylls to carotenoids along with its components had the opposite relationship with the percentage of beta-carotene content to carotenoids of *A. tricolor* genotypes. (Fig. 4).

In this study, we found remarkable pigments profile such as betacyanins, betalains, betaxanthins, and carotenoid profiles such as lutein, violaxanthin, neoxanthin, zeaxanthin, total xanthophylls, beta-carotene in *A. tricolor* genotypes. The results of total carotenoids of our study corroborated with the results of Khanam and Oba^37^ where they observed higher carotenoids in the red amaranth genotype compared to green amaranth. The genotype VA11 and VA16 contained higher lutein, violaxanthin, neoxanthin, zeaxanthin, total xanthophylls, beta-carotene, and total carotenoids compared to the genotype VA14 and VA6. Hence, the carotenoid profiles of *A. tricolor* genotype could be an important parameter for consumers, playing a crucial role in detoxification of ROS in the human body and preventing antiaging and many degenerative human diseases^17,29^. Our result showed that the *A. tricolor* genotype is an excellent source of lutein, violaxanthin, neoxanthin, zeaxanthin, total xanthophylls, beta-carotene, and total carotenoids among leafy vegetables that has important free radical-scavenging activity^19^. *A. tricolor* genotypes VA11 and VA16 had high carotenoid profiles, such as zeaxanthin, lutein, violaxanthin, neoxanthin, total xanthophylls, beta-carotene, and total carotenoids content. The genotypes VA11 and VA16 might be utilized as high-yielding varieties. These genotypes containing abundant carotenoid profiles could be used for extracting colorful juice as drinks, preservatives, and colorant of food products. It revealed from the current investigation that VA11 and VA16 have abundant carotenoids that explore new insight for comprehensive pharmacological study.

### Betaxanthins, betalains and radical scavenging capacity

**Figure 5:**
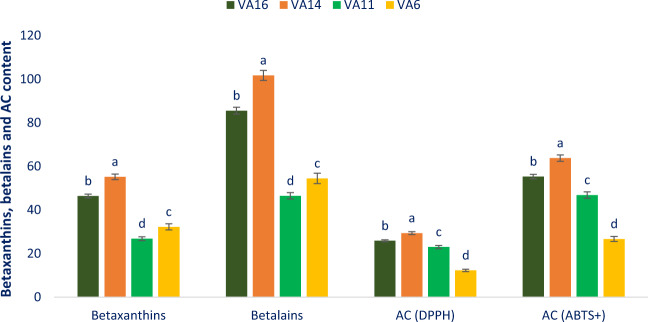
Betaxanthins, betalins, and antioxidant capacity in four selected *A. tricolor* leafy vegetables, betaxanthins (mg 100 g^−1^ FW), betalains (mg 100 g^−1^ FW), AC (DPPH) = Antioxidant capacity (DPPH) (TEAC µg g^−1^ DW), AC (ABTS^+^) = Antioxidant capacity (ABTS^+^) (TEAC µg g^−1^ DW), different letters in the blue and green bars are differed significantly by Duncan Multiple Range Test ((P < 0.01), (n = 3).

Betaxanthins, betalains, and antioxidant capacity (AC) varied significantly among the studied *A. tricolor* leafy vegetable genotypes (Fig. 5). Betaxanthins exhibited much prominent variations regarding genotypes. Betaxanthins varied from 26.87 mg 100 g^−1^ in VA11 to 55.21 mg 100 g^−1^ in VA14. Similarly, betalains varied from 46.47 mg 100 g^−1^ in VA11 to 101.73 mg 100 g^−1^ in VA14. Antioxidant capacity (DPPH) varied from 12.27 µg g^−1^ (VA6) to 29.38 µg g^−1^ (VA14). The maximum antioxidant capacity (DPPH) was noted in VA14 followed by VA16 and VA11. Conversely, VA6 demonstrated the minimum antioxidant capacity (DPPH). Antioxidant capacity (ABTS^+^) varied from 26.69 µg g^−1^ to 63.79 µg g^−1^. *A. tricolor* accession VA14 demonstrated the maximum antioxidant capacity (ABTS^+^) followed by VA16. On the contrary, antioxidant capacity (ABTS^+^) was the minimum in VA6. Our results were corroborated with the findings of Khanam and Oba^37^. They reported greater betaxanthins, betalains, and antioxidant capacity in the red color amaranth compared to green color amaranth. The *A. tricolor* genotype VA14 and VA16 contained higher betaxanthins, betalains, and antioxidant capacity compared to the genotype VA6 and VA11. Hence, these antioxidant constituents of *A. tricolor* genotype could be an important parameter for consumers, playing a crucial role in quenching of ROS, curing many degenerative diseases and preventing anti-aging and^17,29^. Our result revealed that *A. tricolor* contained ample betaxanthins, betalains, and antioxidant potentials among leafy vegetables that have important free radical-scavenging activity^19^.

In this study, we found remarkable color attributes, betacyanin profiles such as amaranthine, iso-amaranthine, betanin, iso-betanin, carotenoid profiles, such as zeaxanthin, lutein, violaxanthin, neoxanthin, total xanthophylls, beta-carotene, total carotenoids, betaxanthins, betalains, and antioxidant capacity in *A. tricolor* genotypes. Our results were corroborated with the findings of Khanam and Oba^37^. They reported greater antioxidant capacity, betacyanins, betaxanthins, betalains, total carotenoids in the red color amaranth compared to green color amaranth. Betacyanin, total carotenoids, antioxidant capacity (ABTS^+^) and antioxidant capacity (DPPH) of current investigation were corroborated with the findings of *A. tricolor* by Khanam *et al*.^38^. We found 2 to 3 time’s higher β-carotene in red color amaranth compared β-carotene of Raju *et al*.^36^ in *A. gangeticus*. The β-carotene of the leaves of red color amaranths were 2 to 3 times and green color amaranths were twice than the β-carotene of *A. caudatus* leaves^29^. Li *et al*.^39^ in *A. hypochondriacus* leaves noticed the highest quenching capacity of radicals (ORAC and FRAP methods) in comparison to the leaves of *A. caudatus*. They also noted the maximum quenching capacity of radicals (FRAP) in leaves in comparison to seed, stalks, sprouts, and flowers. The genotypes VA14 and VA16 had high color attributes, betacyanin profiles, betaxanthins, betalains, and antioxidant capacity. The genotypes VA14 and VA16 containing high antioxidant profiles and high quenching capacity of radicals might be utilized as high-yielding varieties. It revealed from the current investigation that quenching capacity of radicals have high possibility to feed the community deficient in antioxidants. These genotypes also could be used for extracting colorful juice as drinks, preservatives, and colorant of food products.

### The correlation coefficient study

Correlation of betacyanins, betaxanthins, betalains, and antioxidant capacity of *A. tricolor* leafy vegetables are shown in Table 3. Total betacyanins and betaxanthins, betalains had highly significant positive associations among themselves, with total carotenoids, antioxidant capacity (DPPH and ABTS^+^). It revealed that total betacyanins betaxanthins, and betalains, exhibited strong antioxidant activity. Total xanthophylls, total carotenoids, and β-carotene had positive and significant interrelationships with β-carotene, antioxidant capacity (ABTS^+^ and DPPH), and total carotenoids which revealed high quenching capacity of radicals of major carotenoids. The findings of current investigation were corroborated with the results of our earlier study of drought and salt-stressed *A. tricolor*^24^.

**Table 3:** The correlation coefficient for total betacyanin, major carotenoid components, antioxidant constituents, and antioxidant capacity in four selected *A. tricolor* leafy vegetables.

	Betaxanthins (mg 100 g^−1^ FW)	Betalains (mg 100 g^−1^ FW)	Total xanthophyll (mg 100 g^−1^ FW)	β-Carotene (mg 100 g^−1^ FW)	Total carotenoids (mg 100 g^−1^ FW)	AC (DPPH) (TEAC µg g^−1^ DW)	AC (ABTS^+^) (TEAC µg g^−1^ DW)
Total betacyanin	0.93**	0.96**	0.38	0.47	0.56*	0.78**	0.77**
Betaxanthins		0.94**	0.28	0.42	0.62*	0.88**	0.78**
Betalains			0.32	0.44	0.65*	0.79**	0.86**
Total xanthophyll				0.76**	0.84**	0.95**	0.88**
β-Carotene					0.94**	0.76**	0.85**
Total carotenoids						0.88**	0.87**
AC (DPPH)							0.96**

AC (DPPH) = Antioxidant capacity (DPPH), AC (ABTS^+^) = Antioxidant capacity (ABTS^+^), *significant at 5% level, **significant at 1% level, (n = 3).

In conclusions, we identified betacyanin profiles, such as amaranthine, iso-amaranthine, betanin, iso-betanin, carotenoid profiles, such as zeaxanthin, lutein, violaxanthin, neoxanthin, total xanthophylls, beta-carotene, and total carotenoids, betaxanthins, betalains, and antioxidant potentiality (DPPH and ABTS^+^) in *A. tricolor* genotypes. *A. tricolor* genotype VA14 and VA16 had abundant betacyanin components, betaxanthins, betalains and antioxidant potentiality might be utilized as high-yielding varieties. VA11 and VA16 had abundant carotenoids components might be utilized as high-yielding varieties. It revealed from the correlation study that all phytopigments of *A. tricolor* exhibited good quenching capacity of radicals. It also revealed from the current investigation that these accessions had ample antioxidants to quench ROS and had high possibility to study pharmacologically in details. These genotypes also could be used for extracting colorful juice as drinks, preservatives, and colorant of food products. The data on color attributes, betacyanins, carotenoids, betaxanthins, betalains and antioxidant potentiality obtained in the present study could contribute to the scientists for scientific evaluation of these active compounds in *A. tricolor*.

## Methods

### Experimental materials

We selected four high yields and antioxidant potential genotypes from 43 genotypes. It is the first report on color attributes, betacyanin and carotenoid profiles, and antioxidant potentials in *A. tricolor*.

### Design and layout

We executed the experiment in three replicates following a completely randomized block design (RCBD) at Bangabandhu Sheikh Mujibur Rahman Agricultural University. Each genotype was grown in 1 m^2^ experimental plot following 20 cm and 5 cm distance between rows and plants, respectively.

### Intercultural practices

Recommended fertilizer doses, compost, and adequate cultural management were practiced^40^. During final land preparation, total compost (10 ton/ha), triple superphosphate, murate potash and gypsum fertilizers were applied. Whereas, urea was applied in three equal installments i. e., during final land preparation, 10 days after sowing of seed (DAS) and 20 DAS. The urea, triple superphosphate, murate potash and gypsum fertilizers were applied at the rate of 200, 100, 150, and 30 kg/ha, respectively. As the compost was made from rotten cow dung and rice straw, it released the nutrient evenly throughout the experiment period. It also helped to hold the nutrients of chemical fertilizers and released these nutrients evenly. For maintaining the exact spacing of plants in a row, proper thinning was executed. Weeds of experimental plots were regularly removed through proper weeding and hoeing. We provide regular irrigation in the experimental plots for maintaining the proper growth of vegetable amaranth. We collected the leaf samples at 30 days old plant.

### Solvent and reagents

Solvent: acetone, hexane, and methanol. Reagents: acetonitrile (HPLC grade), dichloromethane, and acetic acid, betanin, beta-carotene (98%), lutein (99%), neoxanthin (95%), violaxanthin (98%), zeaxanthin (98%), phenolic acids, and flavonoids, 2, 2-dipyridyl, ABTS^+^, DPPH (2, 2-diphenyl1-picrylhydrazyl), ammonium acetate, petroleum ether, sodium chloride, sodium sulphate, Trolox (6-hydroxy-2, 5, 7, 8-tetramethyl-chroman-2-carboxylic acid), potassium persulfate, sodium carbonate, aluminum chloride Folin-Ciocalteu reagent, hexahydrate, potassium acetate, quercetin, and salicylic acid.

### Determination of color attributes

We measured the color attributes C*, L*, b*, and a* using a TES-135A Plus color meter (Taiwan) in fifteen replicates. Positive b value (+b*) represents yellowness, while negative b value (−b*) indicates blueness. Positive a value (+a*) indicates the magnitude of redness, while negative a value (−a*) indicates magnitude of greenness. L* represents lightness and C* value represents magnitude of leaf color designated as chroma. The chroma value was estimated following the formula, Chroma C* = (a^2^ + b^2^)^1/2^.

### Extraction of samples for LC-MS and HPLC analysis

Ten ml methanol (80%) containing acetic acid (1%) was added in leaf sample (1 g). The mixture was thoroughly homogenized and transferred to a 50 ml tightly capped test tube. The test tubes were placed in a Scientific Industries Inc. shaker (USA) for fifteen h at 400 rpm. A MILLEX^®^-HV syringe filter, USA (0.45 µm) was utilized to filter the mixture. It was centrifuged for fifteen min at 10,000 × g. Betacyanin components were detected from this filtrate. Betacyanin analysis in the samples could interfere through precipitation of methanol with the proteins and other insoluble substances in the samples. A Polymeric Reversed-Phase cartridges (33 µ) (Strata^TM^-X, USA) was utilized to purify betacyanin. The samples were extracted in triplicates.

### Betacyanin analysis through HPLC

The method previously described by Stintzing *et al*.^33^ was followed to determine betacyanin components in *A. tricolor* leaf sample using HPLC. The high performance liquid chromatograph Shimadzu SCL10Avp, Kyoto, Japan was furnished with a degasser (DGU-14A), an LC-10Avp binary pumps, and a Shimadzu SPD-10Avp detector (UV–vis). A STR ODS-II, 150 × 4.6 mm I.D., CTO-10AC column (Shinwa Chemical Industries, Ltd., Kyoto, Japan) was utilized to detected the betacyanin components. The binary mobile phase was pumped with solvent A (acetic acid 6% v/v) in water and solvent B (acetonitrile) at the flow rate of 1 ml/min for seventy min. High performance liquid chromatograph was run using a gradient program containing acetonitrile 0–15% for forty five min, 15–30% for fifteen min, 30–50% for five min and 50–100% for five min. Exactly 35 °C temperature was continued in the column with 10 μl injection volume. The betacyanin was simultaneous monitored by setting the detector at 538 nm. The betacyanin components were detected through comparing UV–vis spectra and retention time with their respective standard compounds. We confirmed the betacyanin components through the method of mass spectrometry. The betacyanin components were detected in triplicates. The betacyanin components were estimated as mg 100 g^−1^ of fresh weight. The LC-MS analysis was done using a mass spectrometer (Tokyo, Japan) equipped with HPLC (Agilent 1100 Series) and a detector coupled with on-line ElectroSpray Ionization (ESI) source. The LC-MS analysis was done in negative ion mode with the needle voltage at −2000 V and the range of the column elutes was m/z 0–1000. Extract constituents were identified by LC-MS-ESI analysis.

### Quantification of betacyanin components

Calibration curves of the respective standards were used to quantify individual phenolic compounds. The betanin was dissolved in methanol (80%) to prepare the stock solutions of 10, 20, 40, 60, 80, and 100 mg/ml. A standard curves were made from these solutions to quantify the individual betacyanin components with external standards. The retention times, co-chromatography, and UV spectral characteristics of samples spiked with available standards (commercially) to identify and match the betacyanin components. Betanin standard was used to prepare standard curves based on the equimolecular conversion for estimating amaranthine and iso-amaranthine in the different samples.

### Sample preparation for extraction of carotenoids

The fresh leaves were thoroughly washed and dried using blotting papers. All precautions were taken to protect photo-isomerization and photo-oxidation and prevent any losses of carotenoids. Sampling was performed at 20 °C with subdued lighting. We ground the dried leaves using a mechanical blender. The powdered leaves were kept in a bag (self-sealing) containing aluminum foil inside the bag at below −20 °C.

### Extraction of carotenoids

We extracted carotenoids following the procedure of Lakshminarayana *et al*.^41^. We extracted carotenoids with acetone (ice-cold) until the powdered leaves became colorless. To prevent oxidation of carotenoid rapid extraction in acetone (ice-cold) was performed. Petroleum ether (100 ml) and 25% aqueous sodium chloride (w/v) (100 ml) were added to leaf extract (50 ml) in a funnel. After mixing, we separated the upper layer. We repeated it for three times (final volume: 250 ml). We dried the extract over 20 g anhydrous sodium sulphate and filtered through a filter paper (Whatman No.1). A rotary evaporator was used to evaporate the filtrate at 35 °C to dryness. We re-dissolved the dried filtrate in hexane (known volume) and an aliquot (100 µl) for analyzing it using high performance liquid chromatograph. We carried out homogenization, extraction, and sample handling under dim yellow light at 4 °C to reduce photo-oxidation and photo-isomerization of carotenoids.

### HPLC analysis

We estimated the carotenoid profiles in *A. tricolor* leaf sample using high performance liquid chromatograph (Shimadzu SCL10Avp, Kyoto, Japan)^41^. A variable detector, binary pumps, and a degasser were equipped with the HPLC system. Briefly, the carotenoids were separated on a column (STR ODS-II, 150 × 4.6 mm I.D., Kyoto, Japan). The carotenoids were separated using methanol:acetonitrile:dichloromethane with a ratio of 20:60:20 (v/v/v) containing ammonium acetate (0.1%) as a mobile phase. For HPLC analysis, leaves (20 µl) were injected under the isocratic condition at a flow rate of 1 ml/min. The detector was set at 450 nm. The SPD-10AVP detector were equipped with Shimadzu model LC-10Avp series to confirm the peak of carotenoids in comparison with their retention time of standard chromatograms. While the characteristic spectrum record with a PDA detector was taken to confirm the λ_max_ values of these compounds. We quantified the carotenoid profiles estimating the area of peak of corresponding standards.

### Betaxanthins content measurement

The leaves of *A. tricolor* leafy vegetables were extracted in methyl alcohol (80%) containing ascorbate (50 mM) to measure betaxanthins following the method of Sarker and Oba^42^. A Hitachi, U-1800 spectrophotometer (Tokyo, Japan) was utilized to read the absorbance at 475 nm for betaxanthins. We calculated the betaxanthins as miligrams per gram of fresh weight equivalent to indicaxanthin.

### Antioxidant capacity assay

Thirty days old *A. tricolor* leaves were harvested. Antioxidant capacity assay, the leaves were dried in the air in a shade. Aqueous methanol (40 ml, 90%) was utilized to extract grounded dried leaves (1 g) from each cultivar in a capped bottle (100 ml). A Thomastant T-N22S (Japan) shaking water bath was utilized to extract leaf samples for 1 h. A MILLEX^®^-HV syringe filter (0.45 µm) (USA) was used to filter the homogenized mixture. After centrifugation for fifteen min at 10,000 × g, the antioxidant capacity was estimated from the extract.

Diphenyl-picrylhydrazyl (DPPH) radical degradation method^43,44^ was used to estimate the antioxidant activity. We added DPPH solution (250 µM, 1 ml) to extract (10 µl) in a test tube (in triplicate). Finally we added 4 ml distilled water the extract and kept in the dark (30 min). A spectrophotometer (Hitachi, Tokyo, Japan) was used to take the absorbance at 517 nm. The antioxidant activity was also estimated following ABTS^+^ assay^45,46^. To prepare two stock solutions separately ABTS^+^ solution of 7.4 mM and potassium persulfate of 2.6 mM were used. We mixed both solutions in equal proportion to prepare the working solution at room temperature. The working solution was allowed to react in the dark for 12 h. One hundred fifty μl extract was added to 2.85 ml of ABTS^+^ solution and allowed to react in the dark for 2 h. For the preparation of the solution, one ml of ABTS^+^ solution was mixed with sixty ml of methanol. A spectrophotometer (Hitachi, Tokyo, Japan) was utilized to take the absorbance at 734 nm. The inhibition of ABTS^+^ (%) and DPPH (%) with control were utilized to calculate quenching capacity of radicals using the equation:

$${\rm{Antioxidant}}\,{\rm{activity}}\,( \% )=({\rm{A}}.\,{\rm{blank}}-\,{\rm{A}}.\,{\rm{sample}}/{\rm{A}}.\,{\rm{blank}})\times 100$$

Where, A. sample is the absorbance of the test compound and A. blank is the absorbance of the control reaction [10 µl methanol (DPPH), 150 μl methanol (ABTS^+^) instead of extract]. The quenching capacity of radicals were calculated as μg g^−1^ of dry weight equivalent to Trolox.

### Statistical analysis

Statistix 8 software was used to analyze the data for analysis of variance (ANOVA)^47^. Duncan’s Multiple Range Test (DMRT) at a 1% level of probability was used to compare the means. The results were reported as the mean ± SD of three separate replicates.

### Ethical statement

The lab and field experiments in this study were carried out as per guidelines and recommendations of “Biosafety Guidelines of Bangladesh” published by the Ministry of Environment and Forest, Government of the People’s Republic of Bangladesh (2005).

## Data Availability

Data used in this manuscript will be available to the public.
